# Introduction of generic substitution and reference pricing in Ireland: early effects on state pharmaceutical expenditure and generic penetration, and associated success factors

**DOI:** 10.1186/2052-3211-8-S1-O10

**Published:** 2015-10-05

**Authors:** Susan Spillane, Cara Usher, Kathleen Bennett, Roisin Adams, Michael Barry

**Affiliations:** 1 National Centre for Pharmacoeconomics, St James's Hospital, Dublin, 8, Ireland; 2 Department of Pharmacology and Therapeutics, School of Medicine, Trinity College Dublin, Dublin, 2, Ireland

## Background

In response to the financial crisis and under the EU/IMF Programme of Financial Support, Ireland committed to make savings on pharmaceutical expenditure, including through a system of generic substitution (GS) and reference pricing (RP) [1]. This intervention involved GS based on fifth level ATC code (active substance), which was followed by RP 3-5 months later. The aim of this analysis was to determine estimates of savings to the healthcare payer resulting from the introduction of GS and RP and to examine utilisation patterns pre and post intervention.

## Methods

This study was a retrospective analysis of patient-level national pharmacy claims data from January 2013-October 2014 inclusive. These data were sourced from the publicly-funded Irish community pharmacy drug reimbursement schemes. For each product deemed interchangeable for GS, average prices over the prior 6 months were calculated (‘pre-price’). Pre-prices were then assigned to claims records for the product over the 6 months following RP. Actual ingredient cost expenditure and expected expenditure based on pre-prices were compared and total savings were calculated. Trends in generic versus proprietary product uptake were also monitored.

## Results

Forty-one product types representing 15 active substances (fifth level ATC) underwent GS and RP between August 2013 and May 2014, resulting in a minimum of 6 months follow-up. For the scheme serving the majority of public patients, GS/RP accounted for an overall combined relative decrease in ingredient cost expenditure of 53% in the 6 months following implementation, amounting to combined 6-month savings of over EUR 35million (total ingredient cost expenditure on these drugs following GS/RP: EUR 31m). Greatest savings were observed for the drug atorvastatin, which incurred a 71% expenditure drop, while lowest relative savings were observed for the drug ramipril, which incurred a 24% expenditure drop. Generic usage rates for the drugs concerned increased on average by 44% with GS/RP introduction.

## Conclusions

The introduction of GS and RP led to substantial savings which corresponded to pre-policy health service official estimates. Compliance with policies is high with generic drug usage targets now exceeded. Success factors included enactment of supportive legislation in 2013, acceptability of active-substance based GS and the phased nature of the policy introduction. On-going work will identify non-compliance with the policies and will aim to elucidate factors affecting the same. The long-term impact of the policies has yet to be determined.

## References

[B1] Economic Adjustment Programme for Ireland Autumn 2012 Review [Internet] 2013 European Commission [cited 2015 May 25]. Available from: http://ec.europa.eu/economy_finance/publications/occasional_paper/2013/pdf/ocp127_en.pdf

